# Nanoencapsulation of sulforaphane in broccoli membrane vesicles and their *in vitro* antiproliferative activity

**DOI:** 10.1080/13880209.2021.1992450

**Published:** 2021-10-29

**Authors:** Lucía Yepes-Molina, Micaela Carvajal

**Affiliations:** Aquaporins Group, Centro de Edafología y Biología Aplicada del Segura (CEBAS-CSIC), Murcia, Spain

**Keywords:** Anticancer activity, Brassicas, encapsulation, isothiocyanates, melanoma, nanocarrier

## Abstract

**Context:**

The development of nanocarriers of plant origin, such as plant cell membranes, has recently been investigated. Also, plant bioactive compounds as sulforaphane (SFN) from broccoli have recognized antioxidant or anticancer properties.

**Objective:**

To investigate the capacity of membrane vesicles from broccoli (BM-vesicles) to encapsulate SFN and their application in the cancer cell line.

**Materials and methods:**

Physicochemical analysis was carried out to characterize BM-vesicles through different approaches: dynamic light scattering, transmission electron microscopy, stopped-flow analysis, and proteomic analysis. They were applied at different concentrations (BM-vesicles at 0.04–0.00315% of protein and SFN at 5, 25, and 100 µM) in SK-MEL-28 cells during 24 h for studying cytotoxicity and gene expression.

**Results:**

The entrapment efficiency was 41%. The anticancer activity tested in cells showed a decrease in proliferation when SFN in BM-vesicles was utilized. Expression patterns when SFN was applied in an encapsulated form showed a reduction of cancer markers and an increase of AQP3. Also, the metabolism of SFN occurred inside of cells, and higher SFN penetrated when it was encapsulated.

**Discussion:**

The results showed that encapsulated SFN was better absorbed by melanoma cells providing metabolism products and a reduction of cancer molecular markers. Also aquaporin, AQP3 was pointed to as an important marker since it appeared to play a key role in homeostasis due to the importance of water transport in biological processes.

**Conclusion:**

These results indicate that SFN and SFN encapsulated in BM-vesicles have a high activity for the inhibition of melanocyte development. Therefore, BM-vesicles could serve as nanocarriers for drugs.

## Introduction

In the past decades, nanocarriers have been highlighted for their great potential in the improvement and development of multiple biotechnological applications, due to their efficient transport of drugs or bioactive compounds (Allen and Cullis 2004). Different types of nanocarriers have been developed and tested in several biotechnological applications: silica, metallic, or carbon-based nanoparticles (NPs), such as inorganic NPs, and lipid or polymer-based NPs, which are classified as organic NPs (Rout et al. 2018). These organic NPs are characterized by being composed of organic compounds, such as lipids and polymers ranging in diameter from 10 nm to 1 µm (Rout et al. 2018; Lombardo et al. 2019). Organic nanocarriers are characterized by high biocompatibility and a very powerful drug loading capacity, whether hydrophilic or hydrophobic drugs (Fattal et al. 2012; Lombardo et al. 2019).

Included in this type of nanoparticles are proteoliposomes, which are formed by lipids and proteins (Lu et al. 2018; Martínez Ballesta et al. 2018). The added value of proteoliposomes over liposomes is that the incorporation of functional proteins grants them transfer properties, such as efficient intracellular delivery, improved circulation, or increased targeting (Lu et al. 2018). Furthermore, the proteins provide the liposomes with additional stability due to the specific lipid-protein interactions which simulate a lipid-protein environment similar to native membranes (Martínez Ballesta et al. 2016; Seneviratne et al. 2018). Proteoliposomes can be synthesized *in vitro* (Lu et al. 2018) or can be obtained from natural sources (Martínez Ballesta et al. 2018).

Recently, several research studies have focussed on membrane vesicles derived from different natural sources, including plants, as new and promising proteoliposome nanocarriers for use in biotechnological applications, such as therapy or cosmetic applications (Ju et al. 2013; Wang et al. 2013; Yepes-Molina et al. 2020, 2021). These types of vesicles are considered to have a high biodegradability and biocompatibility due to their similarity in composition with mammalian exosomes (Ju et al. 2013). Some studies have already shown the potential of this type of nanocarriers. Grape-derived exosome-like NPs promoted the proliferation of stem cells and help to regenerate intestinal epithelial tissue (Ju et al. 2013). Grapefruit-derived nanovectors were used successfully to inhibit tumour growth in murine cancer models (Wang et al. 2013).

Furthermore, our previous studies have highlighted the advantages of membrane vesicles derived from plants, specifically from the family Brassicaceae, and their successful use in different biotechnological applications (Rios et al. 2019, 2020; Yepes-Molina et al. 2020, 2021). On the one hand, these types of vesicles are thermodynamically stable (Chalbi et al. 2015), have a long shelf-life (Silva et al. 2007; Martínez Ballesta et al. 2018), and an industrial application of these vesicles would take advantage of surplus *Brassica* crop biomass (Domínguez-Perles et al. 2010). On the other hand, studies with membrane vesicles derived from *Brassica oleracea* L. var. *Italica* (broccoli) showed its ability to stabilize bioactive compounds, such as glucosinolate glucoraphanin (Martínez Ballesta et al. 2016), to fuse with human cells, specifically keratinocyte cells, and to penetrate into the porcine skin layer (Yepes-Molina et al. 2020). Recent work with membrane vesicles derived from *B. oleracea* L. var. *botrytis* (cauliflower), which were characterized previously (Garcia-Ibañez et al. 2021), showed the possibility of using membrane vesicles to successfully encapsulate bioactive compounds, such as plant extracts, with high stability. Also in this work, the system was applied to keratinocyte culture cells, revealing their non-cytotoxicity and protection against oxidative stress (Yepes-Molina et al. 2021).

Vegetables from the Brassicaceae family have been described to promote beneficial effects for human health. Thus, in addition to obtaining membrane vesicles, this family of plants is suitable for obtaining bioactive compounds. These positive human health effects have been attributed to glucosinolates (GLSs), which are almost Brassica-exclusive secondary metabolites, and isothiocyanates (ITCs) (Dinkova-Kostova and Kostov 2012; Prieto et al. 2019), which are produced by the hydrolysis of GLSs, catalyzed by the myrosinase enzyme (EC 3.2.1.147) (Grubb and Abel 2006). The most studied ITC is sulforaphane (SFN) (*R-*1-isothiocyanato-4-methylsulfinyl butane), which is an enzymatic product from the reaction between myrosinase and the glucopharanin GLSs. SFN has been isolated from broccoli and other cruciferous vegetables and has been described to play an anticancer role in many types of cancer, such as melanoma, aside from its participation in antioxidation and detoxification reactions (Fimognari and Hrelia 2007; Hamsa et al. 2011; Arcidiacono et al. 2018; López-Chillón et al. 2019; Soundararajan and Kim 2018). The anti-carcinogenic properties of SFN are related to the induction of phase II carcinogen detoxification enzymes via the Nrf2 pathway (Misiewicz et al. 2004; Houghton et al. 2016; Su et al. 2018). In addition, SFN induces apoptosis, cell cycle arrest, and avoids the metastasis progression of many cancer types (Hamsa et al. 2011; Feitelson et al. 2015). The molecular mechanisms by which SFN acts against the development of tumours are being presently elucidated in several studies, including a RNA-seq study with melanoma cells at different points of treatment with SFN (Arcidiacono et al. 2018). It was shown that SFN induced a reduction in cell proliferation markers, such as metalloproteinases (Thejass and Kuttan 2006), inhibited cell viability by reducing AKT phosphorylation, and induced apoptosis through the activation of some genes, such as caspases 3 and 9, BAX, p53 (Choi and Singh 2005; Hamsa et al. 2011; Rudolf et al. 2014; Arcidiacono et al. 2018) and the downregulation of others, such as NF-κβ and Bcl2 (Hamsa et al. 2011; Arcidiacono et al. 2018).

As for skin cancers and other diseases, these are often difficult to treat. Thus, the application of different and new strategies to reduce, for example, melanoma, is necessary. Melanoma causes more than 75% of the deaths due to skin cancers, and the incidence of this type of cancer is increasing at a fast rate worldwide (Miller et al. 2019). The newest therapies seek non-invasive methods, but the use of skin as a route to administrate drugs has two limitations: most of the compounds have a poor skin barrier penetrability, and natural drugs tend to have poor stability. One of these compounds is SFN, which is unstable in different environments (Franklin et al. 2014), to rapidly accumulate in cells and to be metabolized fast (Fimognari et al. 2008). Therefore, the development of suitable drug transport and delivery systems is needed. In fact, the current literature revealed that encapsulation of SFN significantly improves its stability (Zambrano et al. 2019). Previous works have shown promising results in the treatment of skin disorders with encapsulated SFN. Do et al. (2010) revealed that the encapsulation of SFN in a biodegradable matrix (microspheres of bovine serum albumin) enhanced the efficacy and allowed a sustained release into cells. Cristiano et al. (2019) showed an improvement in the anticancer activity of SFN-loaded in ethosomes^®^ (Touitou et al. 2000), as compared to the free SFN tested on SK-MEL-28 cells.

This work investigates broccoli membrane (BM)-vesicles as potential nanocarriers for the delivery of SFN, to propose a new clinical therapeutic treatment for skin cancers and diseases. BM-vesicles were characterized from a physicochemical point of view (i.e., mean size, size distribution, zeta potential, osmotic water permeability, ultrastructural morphology, proteomic characterization, and ITCs content). Also, the *in vitro* anticancer activity of BM-vesicles with SFN was tested in melanoma cell cultures (SK-MEL-28), addressing different parameters, such as the cytotoxicity caused, the change in the gene expression of key genes, and the SFN penetration into cells, and its metabolism.

## Materials and methods

### Plant culture

Broccoli seeds were pre-hydrated with de-ionized water and aerated continuously for 24 h. After this, the seeds were germinated in vermiculite in the dark at 28 °C for 2 days. They were then transferred to a controlled environment chamber with a 16 h light and 8 h dark cycle, with temperatures of 25 and 20 °C, and relative humidity of 60 and 80%, respectively. Photosynthetically active radiation (PAR) of 400 mmol/m^2^ s was provided by Pacific LED, WT 470 C, LED8OS/840 PSD WB L1600 lights (Philips). After 5 days, the seedlings were placed in Hoagland nutrient solution and continuously aerated. After 4 weeks of growth, the leaves were harvested for the isolation of vesicles.

### Broccoli membrane vesicles (BM-vesicles)

The leaves were cut into small pieces before vacuum-filtering, at a 1:1.6 (w/v) ratio, with an extraction buffer (0.5 M sucrose, 1 mM DTT, 50 mM HEPES and 1.37 mM ascorbic acid, at pH 7.5), and supplemented with 0.6% PVP. The mixture was homogenized using a blender, and filtered through a nylon mesh (with a pore diameter of 100 µm). The filtrate was centrifuged at 10,000 *g* for 30 min, at 4 °C. The supernatant was recovered and centrifuged for 35 min at 100,000 *g* and 4 °C, and the pellet obtained was suspended in 500 µL of FAB buffer (5 mM potassium phosphate buffer and 0.25 M sucrose, pH 6.5) for storage at −80 °C. The protein concentration in this microsomal fraction was determined with the Bradford method (Bradford 1976), using bovine serum albumin as the standard. To obtain SFN-loaded BM-vesicles, the drug at different concentrations (5, 25, or 100 µM) was dissolved in FAB buffer and centrifuged BM-vesicles were resuspended in FAB with SFN with vigorous vortexing.

### Particle size, zeta potential, and polydispersity index analysis

Dynamic light scattering (DLS) was used to detect particle size, zeta potential, and polydispersity index (PdI) at a temperature of 20 °C using a ZetaSizer Nano XL (Malvern Instruments, Malvern, UK). Size, PdI, and zeta potential measurements were carried out at the initial time and after 1 week at 4 °C.

### Osmotic water permeability (Pf)

The functionality and viability of the BM-vesicles with and without SFN were checked by measuring the osmotic water permeability (*Pf*) by stopped-flow light scattering. The kinetics of the volume adjustment of the membrane vesicles were followed by 90° light scattering at λex = 515 nm. The measurements were carried out at 20 °C in a PiStar-180 spectrometer (Applied Photophysics, Leatherhead, UK). The samples were diluted 100-fold in a buffer containing 30 mM KCl and 20 mM Tris-Mes, pH 8.3 (90 mOsmol/kg H_2_O). The vesicles were then mixed with an equal volume of the same buffer used for vesicle equilibration but with a sucrose concentration of 540 mM (630 mOsmol/kg H_2_O). This resulted in a 270 mOsmol/kg H_2_O inward osmotic gradient. The hypo-osmotic shock associated with membrane dilution induced a transient opening of the vesicles and equilibration of their interiors with the extravesicular solution. The *Pf* was computed from the light scattering time course, according to the following equation: *Pf* = *k*_exp_ *V*_0_/*A_v_ V_w_ C*_out_; where *k*_exp_ is the fitted exponential rate constant, *V*_0_ is the initial mean vesicle volume, *A_v_* is the mean vesicle surface area, *V_w_* is the molar volume of water, and *C*_out_ is the external osmolarity.

### Ultrastructural analysis of broccoli membrane-vesicles

The ultrastructural analyses of BM-vesicles with and without SFN were performed through transmission electron microscopy. The vesicles were pelleted at 100,000 *g* and chemically fixed with glutaraldehyde (2.5% in 100 mM phosphate buffer, 2 h at 4 °C), osmium tetroxide (1% buffered, 2 h at 4 °C), and tannic acid (1% in deionized water, 30 min at 22 °C). The pellets were then thoroughly rinsed with water, covered with 2% low-melting-point agarose, dehydrated with ethanol and epoxypropane at 22 °C, and then embedded in Epon. The blocks were sectioned on a Leica EM UC6 ultramicrotome, collected on Formvar-coated copper grids, and stained with uranyl acetate followed by lead citrate. The sections were examined using a JEOL 1011 transmission electron microscope with a GATAN ORIUS SC200 digital camera. For each sample, 5–10 ultrathin sections were examined.

### Isothiocyanate content in broccoli membrane-vesicles

Isothiocyanates BM-vesicles were identified using the UHPLC-QQQ-MS/MS method described by Dominguez-Perles et al. (2014). BM-vesicles were disrupted before analyses with 2% SDS and through sonication. After that, the samples were filtered through a 0.2 µm PVDF filter. Data acquisition was performed by using the MassHunter software version B.08.00 (Agilent Technologies, Waldbron, Germany). ITCs content in BM-vesicles was quantified using the cyclo-condensation reaction between ITC and BDT (Zhang 2012), and SFN as the standard. Potassium phosphate buffer pH 8.5, containing 1% Triton X-100 was prepared to lyse the BM-vesicles. The sample (25 μL), 100 μL of buffer, and 125 μL of 8 mM BDT were mixed and heated in a dry bath at 65 °C for 1 h. The solution was cooled to room temperature and the absorbance was measured at 365 nm.

### Broccoli membrane-vesicles entrapment efficiency (EE) percentage

BM-vesicles with SFN were washed in phosphate buffer (0.33 M sucrose, 5 mM potassium phosphate pH 7.8), followed by centrifugation at 100,000 *g* for 30 min, to remove the drug that was not encapsulated. SFN in the supernatant and the pellet resuspended in the same initial volume were analyzed using the BDT method (Zhang 2012) and UHPLC-QQQ-MS/MS method (Dominguez-Perles et al. 2014) as described above.

### Proteomic characterization of broccoli membrane-vesicle

BM-vesicles were incubated overnight in chloroform:methanol (1:4) at −20 °C. Then, samples were centrifuged (12,000 *g*, 20 min, 4 °C) and washed three times with methanol. Then, the pellet was resuspended in 100 μL of 50 mM ammonium bicarbonate (pH 8.3) with 0.01% ProteaseMax (Promega, Madison, WI, USA). After that, the samples were reduced by adding 100 μL of 20 mM DTT at 56 °C for 20 min. Then, alkylation was performed by incubation with 100 μL of 100 mM IAA for 30 min, in the dark at room temperature. The digestion was performed by incubation with 1 μg of trypsin (1:100 w/w) for 3 h, at 37 °C. The samples were dried in a speed vacuum concentrator and resuspended in 20 μL of water/acetonitrile/formic acid (94.9:5:0.1). Then, they were injected into an Agilent Advance Bio Peptide Mapping HPLC, containing a column (2.7 μm × 100 mm × 2.1 mm, Agilent technologies) set at 55 °C, and a dual electrospray (AJS-Dual ESI). The experimental parameters were set in MassHunter Workstation Data Acquisition software (Agilent Technologies, Santa Clara, CA, USA), as described. Martínez Ballesta et al. (2018). The MS data were processed with the Spectrum Mill MS Proteomics Workbench (Agilent Technologies) using Brassicaceae sequences from the Uniprot database (www.uniprot.org) (Bateman 2019). The biological function, metabolic process, and location of the different identified proteins were determined from the Gene Ontology database (The Gene Ontoly Consortium 2019).

### SK-MEL-28 cells culture and treatments applied

SK-MEL-28 melanoma cell line was obtained from the ATCC (American Type Culture Collection, Manassas, VA, USA). The cells were cultured in Eagle's Minimum Essential Medium (EMEM) supplemented with 10% foetal bovine serum (FBS), 1% penicillin-streptomycin, and 1% l-glutamine at 37 °C and 5% CO_2_. The cells were routinely cultured into 75 cm^2^ culture flasks. Sub-culturing was carried out every three days when the cells reached 70–90% confluence. When the cells reached 60–70% confluence, they were washed with PBS buffer (37 °C) and treatments were applied for 24 h. Cells were treated for 24 h with different concentrations of BM-vesicles (0.04–0.000315% protein), and different concentrations of free SFN and encapsulated-SFN (5, 25, and 100 µM). All the images for morphological analysis were captured using an Eclipse TE 2000-U Nikon inverted microscope (Nikon, Kyoto, Japan).

### Cell cytotoxicity assay

The effect of different concentrations of treatments (BM-vesicles, free SFN, and encapsulated-SFN in BM-vesicles) on the viability of SK-MEL-28 cells was determined with the MTT [3-(4,5-dimethylthiazol-2-yl)-2,5-diphenyltetrazolium bromide] assay (Mosmann 1983). Cells were plated at 5000 cells/well in 198 µL of EMEM complete medium in a 96-well plate and cultivated at 37 °C and 5% CO_2_ until 60–70% confluence. Afterward, 2 µL of different 100× treatments were added to the wells (each sample was repeated six times) and incubated for 24 h. Cell viability was determined by adding 200 µL of MTT (1 mg/mL in DMEM) after completely removing the medium from the wells, and incubated for 4 h at 37 °C and 5% CO_2_. The MTT solution was removed, and 100 µL of DMSO was added and the plate is shaken. The absorbance was recorded at 570 nm on a microplate reader (BMG Labtechnologies, Fluostar Omega). The cell viability (%) of treated cells was calculated as follow:

(1) $$\mathrm{Cell}\;\mathrm{viability}\;(\%)=\;\frac{({{\mathrm{Abs}}_{570nm})}_{\mathrm{sample}\;}}{({{\mathrm{Abs}}_{570nm})}_{\mathrm{control}\;}}\times \;100$$

### Quantitative real-time RT-PCR

SK-MEL-28 cells were seeded into six-well (148,000 cells/well) until cells reached 60–70% confluence, and treatments (BM-vesicles, free SFN, and encapsulated-SFN in BM-vesicles) were applied for 24 h. Then, RNA extraction from SK-MEL-28 cells was performed by using the NZY Total RNA Isolation kit (NZYtech, Lisboa, Portugal) as indicated by the manufacturer. RNA concentrations were determined with Nanodrop 2000 Spectrophotometer (Thermo Fisher Scientific, Waltham, MA, USA). cDNA was synthesized from 500 ng RNA using the High-Capacity cDNA Reverse Transcription Kit (Applied Biosystems) according to the manufacturer’s protocol. Real-time PCR analysis was performed in an Applied Biosystems QuantStudio 7500 Real-Time PCR system (Thermo Fisher Scientific) in 10 μL volumes, using the 2X Power SYBR Green PCR Master Mix (Applied Biosystems, Carlsbad, CA, USA) with ROX passive reference dye. Volumes and concentrations for SYBR Green reaction mixes were 5 μL SYBR Green reaction mix, 500 nM forward and reverse gene-specific primers (Table 1), and 200 ng DNA template. Amplification conditions were: 2 min at 50 °C, 10 min at 95 °C followed by 40 cycles of 15 s at 95 °C and 1 min at 60 °C. The amplifications were performed on three independent samples for each treatment (biological replicates), and triplicate reactions were carried out for each sample (technical replicates) in 96 well plates. Transcript levels were calculated using the 2^–ΔΔCt^ method (Livak and Schmittgen 2001) and by normalizing to that of the housekeeping gene β-actin.

**Table 1.** Primer sets used for qRT-PCR.

Target name	Forward primer (5′ → 3′)	Reverse primer (5′ → 3′)	References
β-Actin	AAATCTGGCACCACACCTTCTAC	ATAGCACAGCCTGGATAGCAAC	Kostyuk et al. 2018
TNFα	TCCTTCAGACACCCTCAACC	AGGCCCCAGTTTGAATTCTT	Niewiarowska-Sendo et al. 2016
p53	CCTCAGCATCTTATCCGAGTGG	TGGATGGTGGTACAGTCAGAGC	Yang et al. 2019
BAX	TCAGGATGCGTCCACCAAGAAG	TGTGTCCACGGCGGCAATCATC	Jiang et al. 2020
AQP3	CTTGAGCATCCACTGACT	GGGTGAGGGTAGATAGGG	Shin et al. 2017

TNF: tumour necrosis factor; BAX: BCL2 associated X; AQP3: aquaporin 3; BM-vesicles: broccoli membrane vesicles; n.d.: non-detected.

### Absorption and metabolism assay using UHPLC-QqQ-MS/MS

SK-MEL-28 cells were seeded in six-well plates (148,000 cells/well) until the cells reached 60–70% confluence, and the treatments (BM-vesicles, free SFN, and encapsulated-SFN in BM-vesicles) were applied. After 24 h of treatment, the media was collected, and the cells were washed twice with PBS. The cells were incubated for 15 min at −80 °C in 80% methanol. After that, the cells were scraped and centrifuged at 20,000 *g* for 10 min. The supernatants and culture media collected were stored at −80 °C until use. Before analysis, the samples were dried with speed-vacuum, resuspended in miliQ water, and filtered through a 0.2 µm PVDF filter. The analysis of SFN and its derivative (SFN-CYS) in the different cell supernatants and culture mediums was carried out in a UHPLC-QQQ-MS/MS, utilizing the method described by Dominguez-Perles et al. (2014).

### Nile red (NR) assay

BM-vesicles (0.2 mg/mL) were incubated in NR solution (50 µg/mL) to label BM-vesicle lipids. BM-vesicles with NR were washed in phosphate buffer (0.33 M sucrose, 5 mM potassium phosphate, pH 7.8), followed by centrifugation at 100,000 *g* for 30 min to remove the free dye. Labelled BM-vesicles were applied to SK-MEL-28 cell cultures at 60–70% of confluence at a final concentration of 0.002 mg/mL protein and 0.02 µg/mL NR. A fluorescence microscope (Nikon Eclipse TE2000-U, Nikon Instruments Europe B.V. Amsterdam, Netherlands) equipped with a digital camera was used to measure NR fluorescence in the cell culture after 30 min of incubation. The excitation of the sample was carried out with 550 nm green light, and red fluorescence was obtained at 640 nm.

### Statistical analysis

The R software (R Core Team 2018) was used to analyze all the data. When multiple comparisons were performed, the evaluation involved a one-way ANOVA followed by Duncan’s test or Student’s *t*-test. Differences were considered to be significant at *p* < 0.05. All the results are presented as the mean ± SE.

## Results

### Physicochemical characterization of BM-vesicles and encapsulated-SFN in BM-vesicles

#### Size, polydispersity index, zeta potential, and ultrastructural analysis

**Figure 1.**
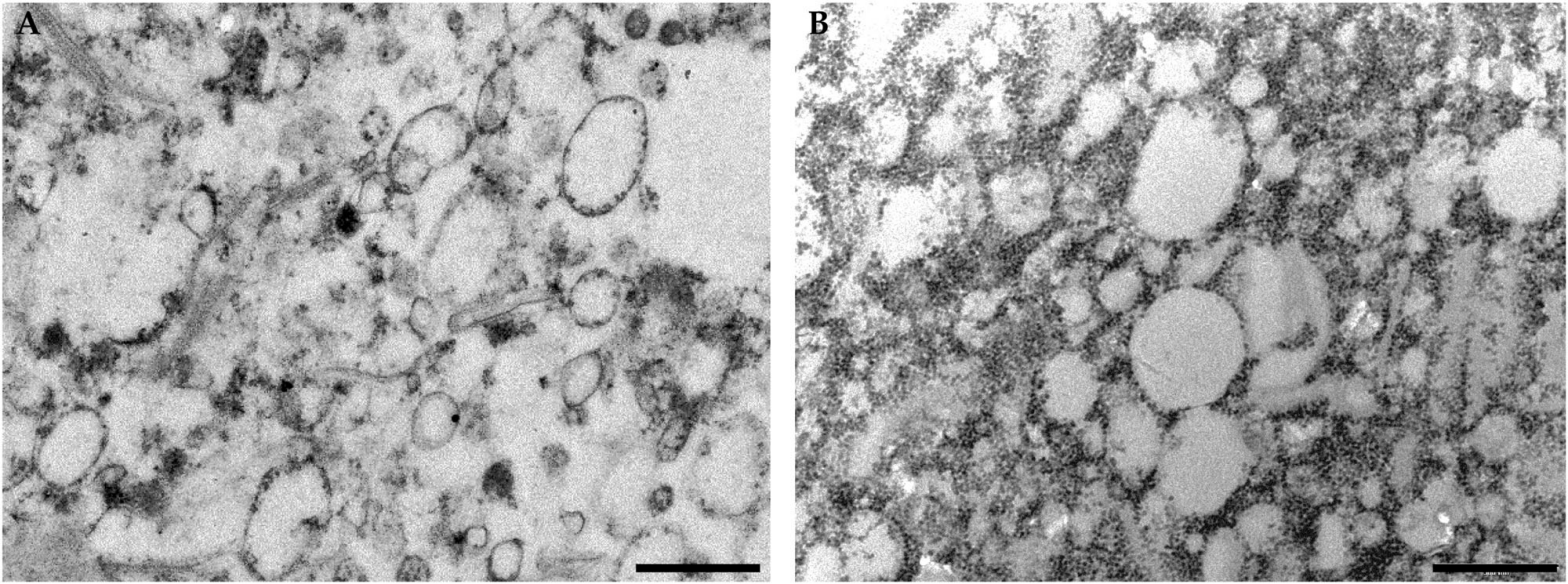
Ultrastructural analysis. Transmission electron microscopy images of BM-vesicles (A) and BM-vesicles with SFN (B). Scale bar = 500 nm.

**Figure 2.**
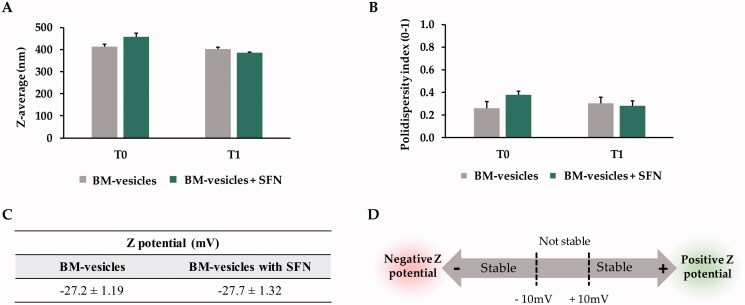
Dynamic light scattering analysis. Z-average (nm) (A), Polydispersity index (B), and Zeta potential (mV) (C) were measured in Broccoli membrane (BM)-vesicles and BM-vesicles with sulforaphane (SFN) at the initial time (T0) and 1 week after sample preparation (T1). Schematic representation of the significance of Zeta Potential values (D). Data are mean ± SE of three independent experiments.

BM-vesicles were spherical in shape when analyzed by transmission electronic microscopy (Figure 1(A)). The spherical shape of BM-vesicles was maintained when SFN was encapsulated in the vesicles (Figure 1(B)). Also, key parameters for vesicles characterization were measured through dynamic light scattering (DLS). BM-vesicles showed a hydrodynamic diameter of around 400 nm, which was stable for 1 week, and this parameter did not change when SFN was encapsulated in BM-vesicles (Figure 2(A)). The polydispersity index (PdI) ranged between 0.2 and 0.3, and significant differences were not observed neither between samples (BM-vesicles with or without SFN) nor between time points (Figure 2(B)). Regarding the Zeta potential, our data indicated that zeta potentials for BM-vesicles with or without SFN were −27 mV (Figure 2(C)).

#### Integrity and functionality

**Figure 3.**
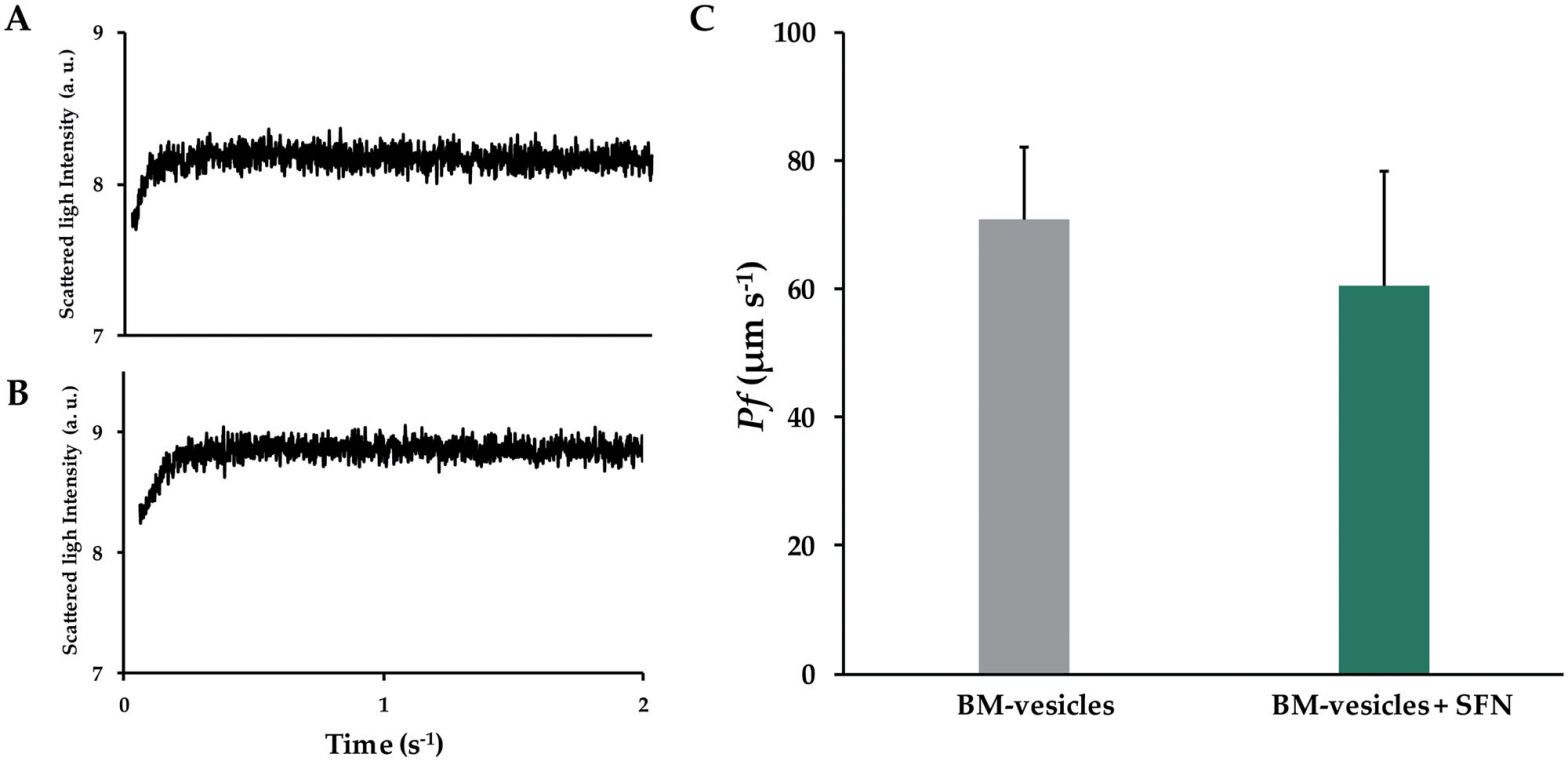
Stopped-flow analysis. Typical traces obtained from stopped-flow spectroscopy after hyperosmotic shock, the signals are shown are representative of ten traces obtained from Broccoli membrane (BM)-vesicles (A), and BM-vesicles with sulforaphane (SFN) (B). Water permeability coefficients (Pf) of BM-vesicles and BM-vesicles with SFN (C). Data are mean ± SE of three independent experiments with at least 10 traces.

The integrity and functionality of BM-vesicles, before and after encapsulating SFN, were evaluated by measuring the osmotic water permeability coefficient (*Pf*) of the vesicles. The shrinking kinetics for BM-vesicles (Figure 3(A)) and BM-vesicles with SFN were similar and with a time-dependent increase in light scattering intensity (Figure 3(B)) that was complete in 0.2 s for both samples. With the osmotic shrinking kinetics and the volume-to-surface ratio, *Pf* values were calculated for both types of samples. Figure 3(C) shows that the *Pf* values of BM-vesicles with or without SFN were not significantly different.

#### Isothiocyanate content

BM-vesicles were obtained from the plant material and their natural content in isothiocyanates was quantified using cycloreactions between ITC and 1,2-benezenedithiol (BDT) (Zhang 2012). The results obtained showed a total ITC content of 0.52 µmoles/mg protein. Furthermore, a complete identification of ITCs was carried out by Ultra High-Performance Liquid Chromatography with Triple Quadrupole type Mass Spectrometer (UHPLC-QqQ-MS/MS). Different isothiocyanates, considered bioactive compounds, were detected in BM-vesicles samples, such as sulforaphane, erucin, iberin, or indole-3-carbinol.

#### Entrapment efficiency (EE)

EE was calculated to know how much SFN was retained inside the vesicles. Ultracentrifugation was used to remove non-encapsulated SFN and the BDT-method and UHPLC-QqQ-MS/MS were used to determine de EE (%). The results obtained revealed an EE of 41.56 ± 8.56%.

#### Proteomic characterization

**Figure 4.**
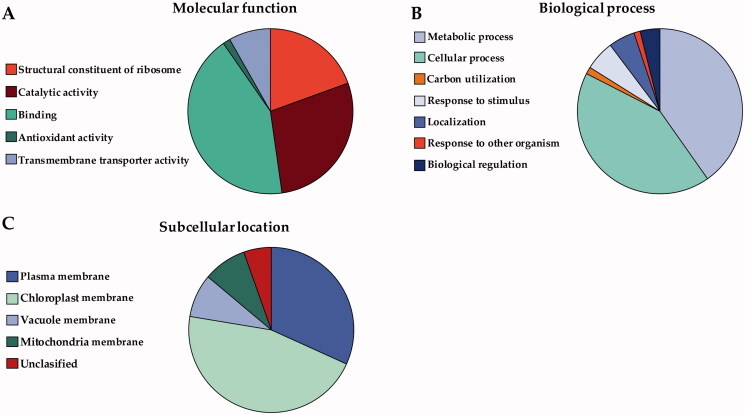
Proteomic analysis. The proteins found in the BM-vesicles were classified according to Molecular function (A), Biological process (B), and Subcellular location (C) according to Gene Ontology (The Gene Ontoly Consortium 2019). The protein data were obtained from the Uniprot database (www.uniprot.org) (Bateman 2019).

A proteomic analysis was carried out to qualitatively evaluate the proteins present in the BM-vesicles. The membrane proteins obtained were organized according to the three Gene Ontology (GO) aspects: Molecular Function, Biological Process, and Subcellular Location (Figure 4).

Membrane proteins were grouped into five Molecular Function clusters (Figure 4(A)). Most membrane proteins identified in BM-vesicles were related with a binding activity (42.59 ± 3.35%), the next most abundant category was related to the catalytic activity (28.29 ± 0.19%), and lastly, membrane proteins associated with the structural constituent of ribosome (19.46 ± 6.49%). In a lesser abundance, we found proteins involved in transmembrane transport (8.21 ± 3.14%), and antioxidant activity (1.44 ± 0.18%).

Regarding Biological processes, membrane proteins of BM-vesicles were classified into seven clusters as shown in Figure 4(B). In this case, there were two major groups: cellular process (42.3 ± 2.02%) and metabolic process (40.22 ± 3.25%). The minority groups (with <5% appearance) into which the membrane proteins found in BM-vesicles were classified, were: response to stimulus, localization, biological regulation, carbon utilization, and response to other organisms.

Lastly, regarding the classification by Subcellular Location (Figure 4(C)), the membrane proteins identified in the BM-vesicles were localized mostly to the plasma membrane (31.77 ± 1.14%) and chloroplast (45.84 ± 2.81%). The rest were found in the vacuole and mitochondria (16.97 ± 0.75%). And to conclude, a small proportion of proteins (5.42 ± 0.91%) could not be classified in terms of subcellular location.

### In vitro antiproliferative activity of BM-vesicles and BM-vesicles encapsulated with SFN in SK-MEL-28 cells

#### Cytotoxic effects in SK-MEL-28 cells of BM-vesicles and BM-vesicles with SFN

**Figure 5.**
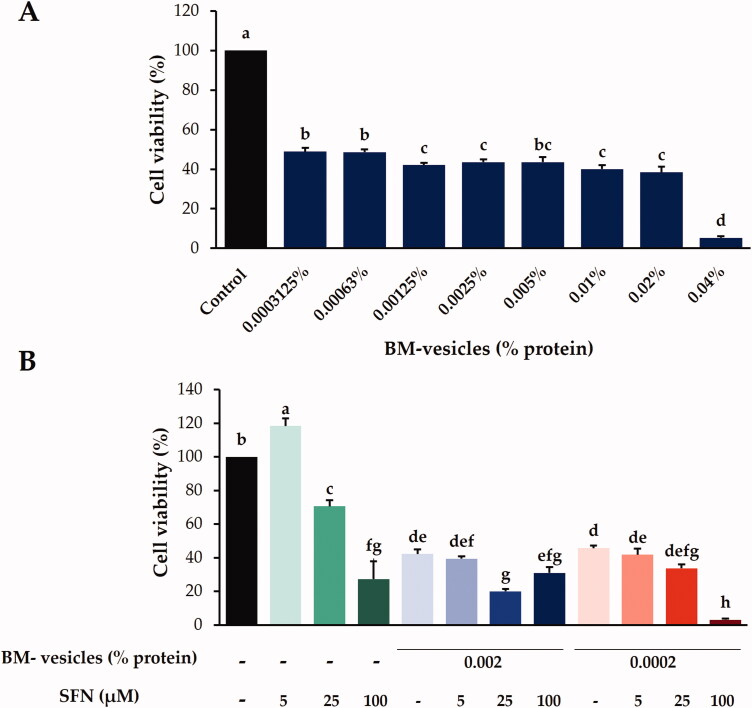
Cytotoxic effects. In the MTT assay, the cytotoxic effects in SK-MEL-28 cells of Broccoli membrane (BM)-vesicles at different concentrations (0.04–0.0003125%) (A) and free and encapsulated sulforaphane (SFN) (5, 25, and 100 µM) (B) were analyzed. Data are mean ± SE of three independent experiments. Different letters indicate significant differences between groups after one-way ANOVA and Duncan’s test (*p* < 0.05).

The cytotoxic effect of BM-vesicles at different concentrations was evaluated with the MTT assay, 24 h after application, in SK-MEL-28 cells (Figure 5(A)). The results did not show a clear dose-dependent increase in toxicity. The highest dose (0.04% protein) showed very potent cytotoxicity, which was significantly different from that caused by the rest of the concentrations. Concentration 0.02–0.0003125% protein showed a cytotoxicity value of around 50%. After that, two concentrations were chosen for the next assays with encapsulated SFN: 0.002 and 0.0002% protein.

The cytotoxicity of SNF-loaded BM-vesicles as compared to free SFN was evaluated both as a function of the drug concentration (5, 25, and 100 µM) with the MTT assay in SK-MEL-28 cells (Figure 5(B)). Free SFN at 5 µM promoted a small increase in cell proliferation, and at 25 and 100 µM, cell viabilities of 70 and 30%, respectively, were observed. Regarding SFN encapsulated in BM-vesicles, we found that when the lowest concentration of SFN (5 µM) was encapsulated at the two concentrations of BM-vesicles tested, no differences were found with respect to cytotoxicity caused by BM-vesicles without SFN. The next concentration of SFN tested, 25 µM, when encapsulated in BM-vesicles (0.002% protein), showed a 20% cell viability, which was significantly less than that promoted by BM-vesicles and much less than that caused by free SFN 25 µM, revealing a synergistic effect between BM-vesicles and SFN, which acted together to decrease cell viability. In general, the cytotoxicity test carried out showed a significant effect on the viability of the SK-MEL-28 cancer cells.

#### Morphological analysis

**Figure 6.**
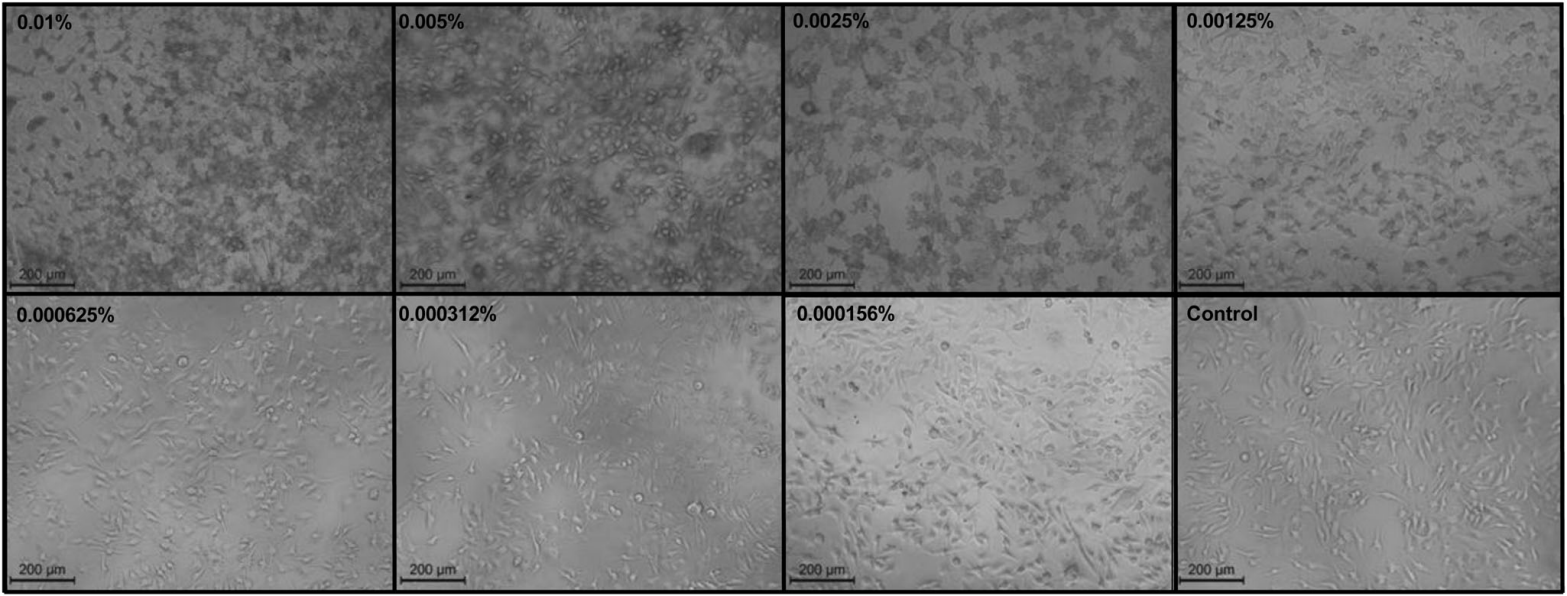
Morphological analysis. Phase*-*contrast microscopy images of SK-MEL-28 cells treated with different concentrations (% protein) of Broccoli membrane (BM)-vesicles. Scale bars = 200 µm.

**Figure 7.**
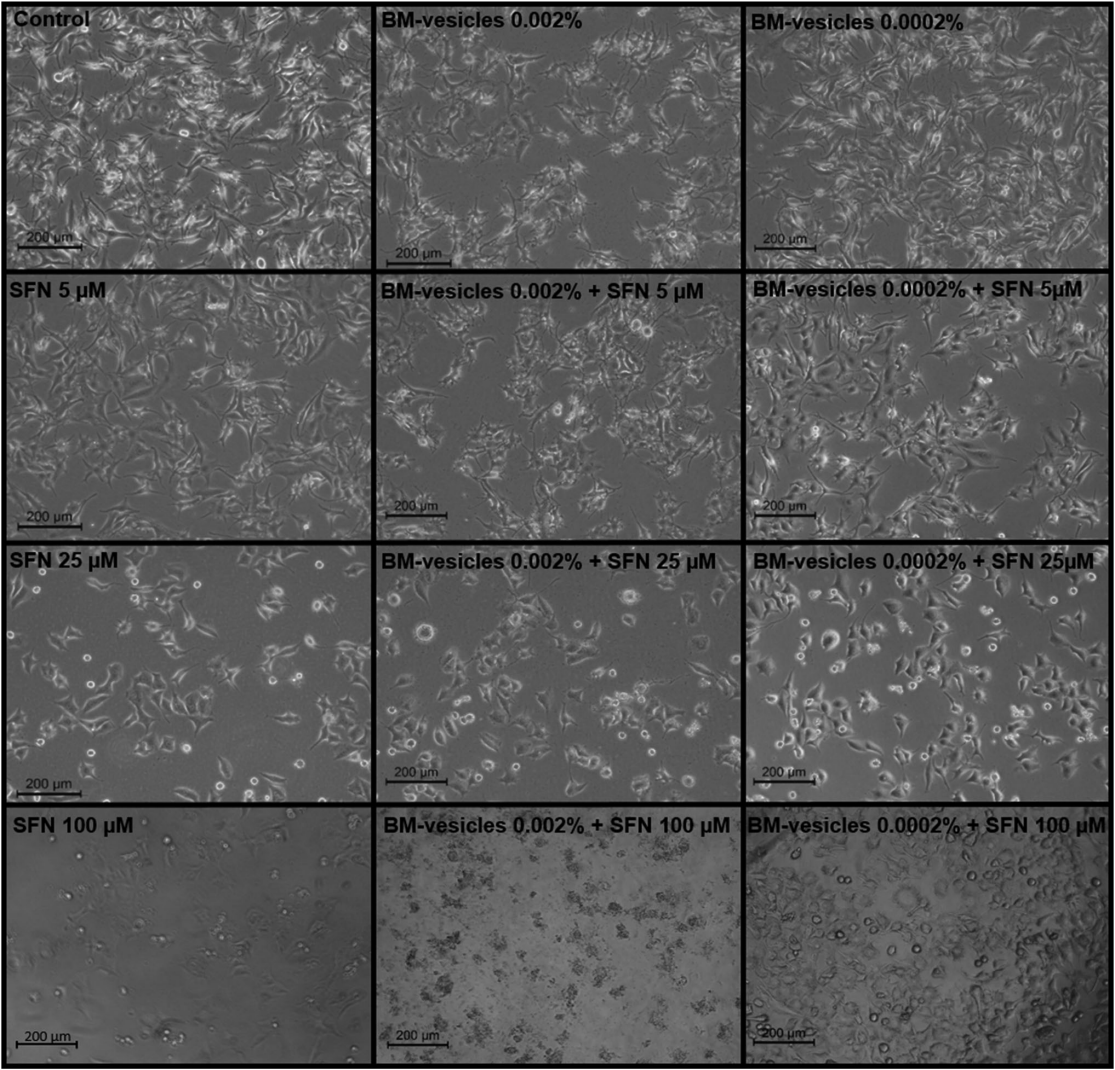
Morphological analysis. Phase*-*contrast microscopy images of SK-MEL-28 cells treated with different treatments: control, free sulforaphane (SFN) (5, 25, and 100 µM), Broccoli membrane-vesicles (BM-V) (0.002 and 0.0002% protein), and SFN (5, 25, and 100 µM) encapsulated in BM-vesicles (0.002 and 0.0002% protein). Scale bars = 200.

The cytotoxic effect of the treatments applied to SK-MEL-28 cancer cells, and evaluated through the MTT assay was also checked visually through phase-contrast microscopy images. Figure 6 shows the morphological changes that could be observed on the cell surface after BM-vesicles application at different concentrations for 24 h. Also, the higher the applied concentration, the lower the visible cell population. At concentrations >0.0025%, a flattening of the cells and a distortion of the cell borders can be observed. The morphological effects of free SFN and encapsulated in BM-vesicles were also checked, as shown in Figure 7. Once again, a loss in the number of cells was observed when the concentrations of SFN were high (25 and 100 µM) or when SFN was encapsulated in BM-vesicles at any concentration. Furthermore, when the free and encapsulated SFN acted on the cells, membrane blebs or a bulge of membrane-bound apoptotic bodies were observed.

#### Effect of BM-vesicles with SFN on the expression of key genes

**Figure 8.**
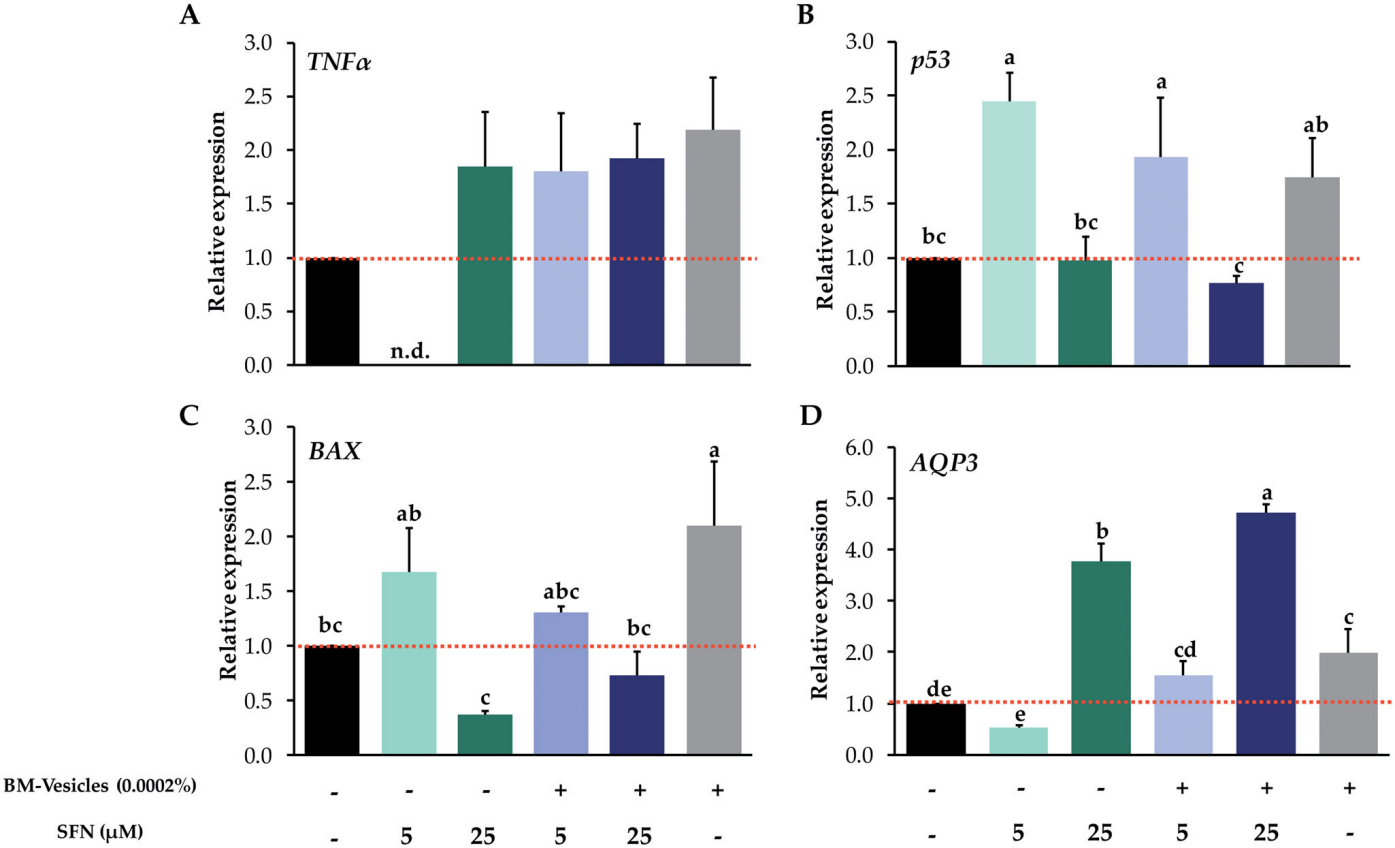
RT-qPCR analysis. Relative gene expression of TNFα (A), p53 (B), BAX (C), and AQP3 (D) in SK-MEL-28 cells after the application of Broccoli membrane (BM)-vesicles (0.0002%) and two concentrations of free or encapsulated sulforaphane (SFN) (5 and 25 µM) for 24 h. Data are mean ± SE (*n* = 3). Different letters indicate significant differences between groups after a one-way ANOVA and Duncan’s test (*p* < 0.05).

The effect of BM-vesicles, BM-vesicles with SFN, and free SFN on the expression of various genes related to apoptosis and proliferation are shown in Figure 8. The expression of TNFα did not show significant differences between the different treatments applied. In addition, when the cells were treated with SFN 5 µM, no expression of this gene was detected by qRT-PCR (Figure 8(A)). Regarding p53 gene expression, this is a pro-apoptotic gene which was up-regulated 2.5-fold after a 24 h incubation of SK-MEL-28 cells with 5 µM SFN, and 5 µM SFN encapsulated in BM-vesicles, while the rest of the treatments did not show significant differences with respect to the control (untreated cells) (Figure 8(B)). BAX, a p53-related gene, was up-regulated in SK-MEL-28 cells treated with BM-vesicles for 24 h, although the other treatments did not cause significant changes in the expression of this gene (Figure 8(C)). Finally, AQP3 expression was also measured by qRT-PCR, and the result showed an up-regulation when cells were treated with 25 µM SFN, 25 µM SFN encapsulated in BM-vesicles, and BM-vesicles to 4, 4.5, or 2-fold, respectively (Figure 8(D)).

#### SFN penetration into cells and metabolism

**Figure 9.**
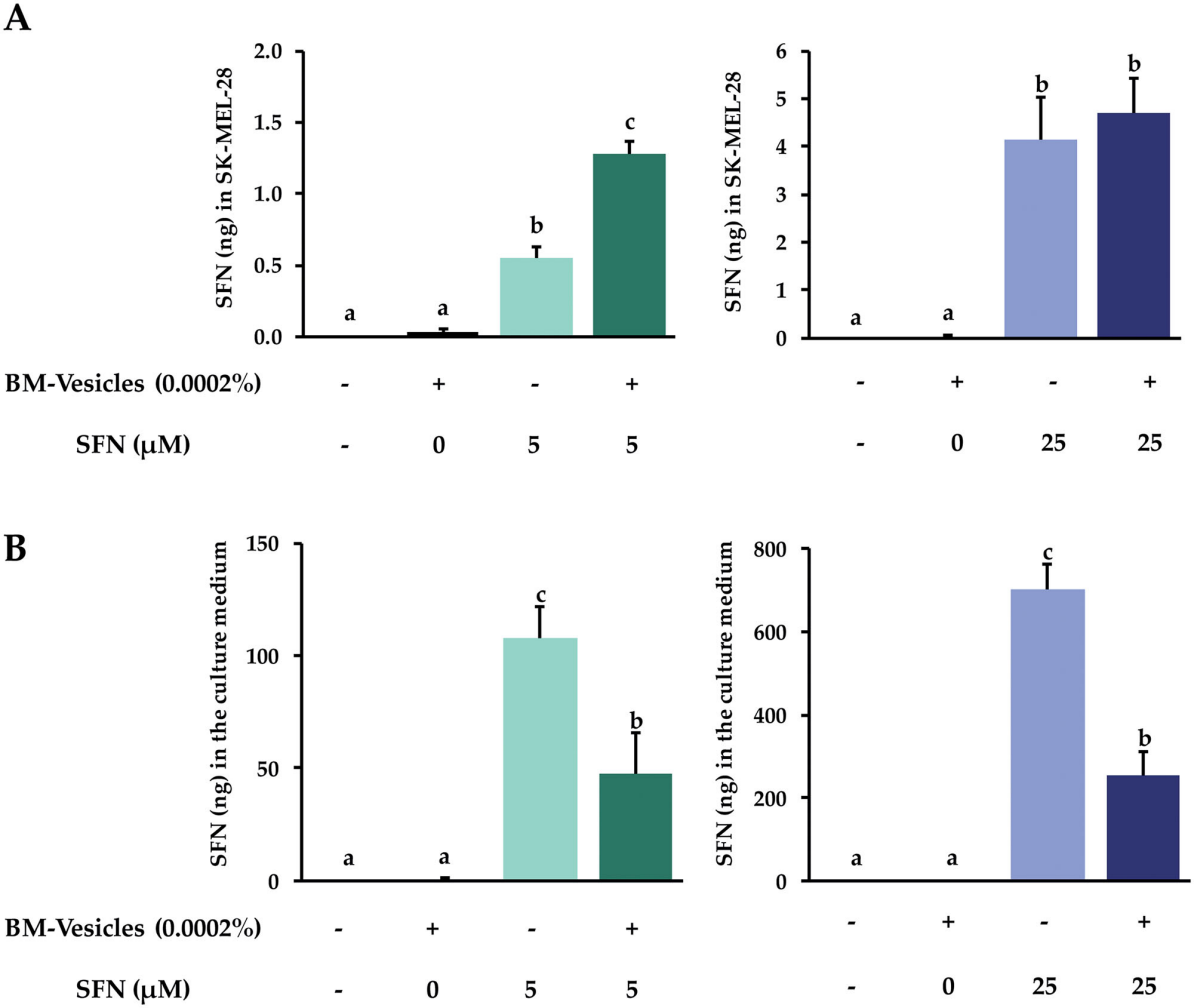
SFN metabolism analysis. Amount (ng) of sulforaphane (SFN) inside SK-MEL-28 cells (A), and their release into the culture medium (B) after the application of Broccoli membrane (BM)-vesicles (0.0002%) and two concentrations of free or encapsulated sulforaphane (SFN) (5 and 25 µM) for 24 h. Data are mean ± SE (*n* = 3). Different letters indicate significant differences between groups after a one-way ANOVA and Duncan’s test (*p* < 0.05).

After 24 h of BM-vesicles and free and encapsulated SFN treatments, different amounts of SFN were found in cell lysates in SK-MEL-28 cells (Figure 9(A)). The treatment with BM-vesicles alone did not allow finding a significant amount of SFN inside cells. When 5 µM of SFN was applied for 24 h, a higher amount of SFN was found in cell lysates when the bioactive compound was applied encapsulated in BM-vesicles, as compared to that found when free SFN was used. However, no significant differences were found in the amount of SFN inside cells when 25 µM of SFN were applied either free or encapsulated in BM-vesicles. On the other hand, Figure 9(B) shows SFN (ng) found in the culture medium after the application of different treatments for 24 h. Similar results were found when 5 or 25 µM of SFN were applied, in the sense that a lower amount of SFN was detected in culture medium when SFN was applied encapsulated in BM-vesicles as compared to that found when applying free SFN.

With the application of 5 and 25 µM of SFN, conjugated metabolites derived from SFN were not detected within the cells. But SK-MEL-28 cell lysates were analyzed after application of free or encapsulated 100 µM SFN in BM vesicles, and sulforaphane-cysteine (SFN-CYS), a conjugate of SFN, was detected (Table 2).

**Table 2.** Metabolite sulforaphane-cysteine (SFN-CYS) was measured in cell lysates in SK-MEL-28 cells after the application of the treatments for 24 h.

Applied treatments	SFN-CYS (ng) in cell lysates
Control	n.d.
SFN 100 µM	8.23 ± 2.19
BM-vesicles + SFN 100 µM	0.83 ± 0.2
BM-vesicles	n.d.

SFN: sulforaphane; BM-vesicles: broccoli membrane vesicles; n.d.: non-detected.

Data are mean ± SE (*n* = 3).

#### BM-vesicles penetration into cells

**Figure 10.**
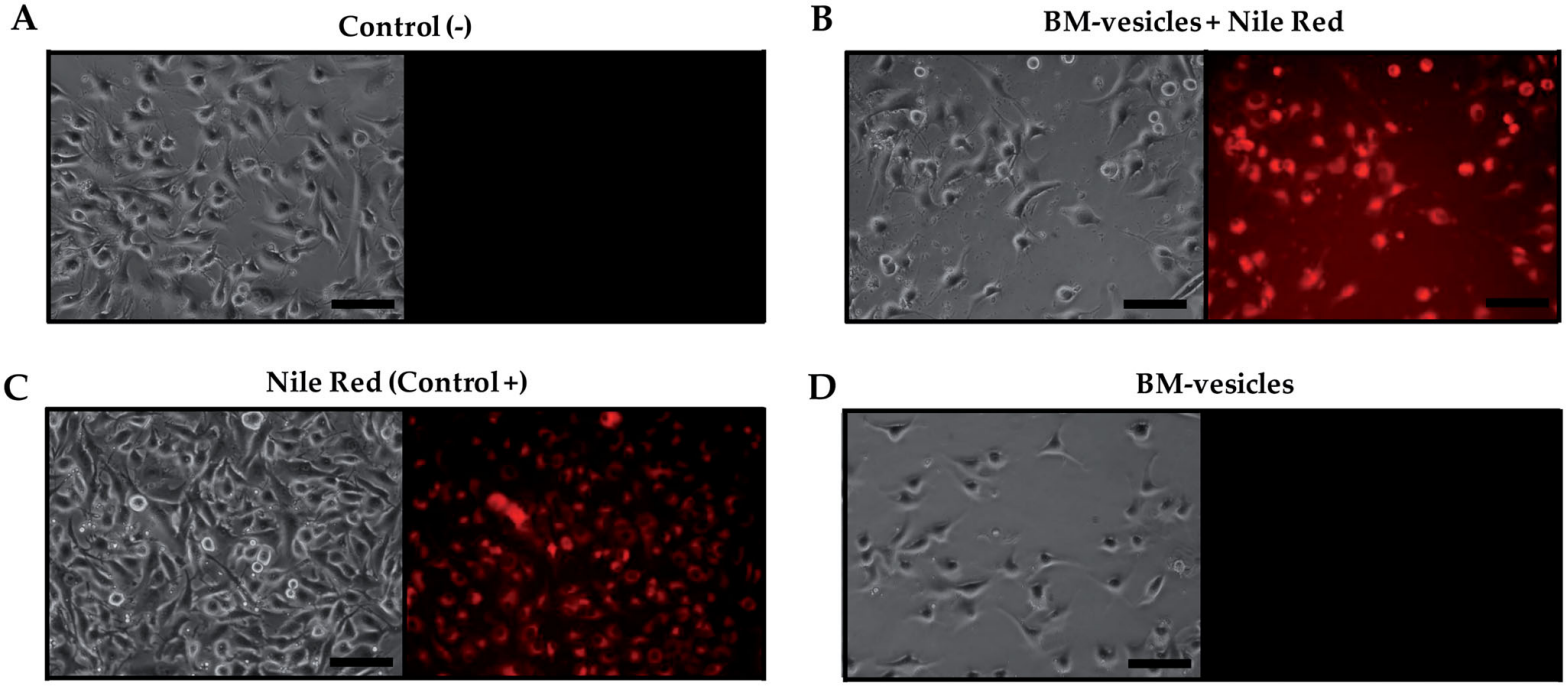
BM-vesicles penetration in cells. Phase-contrast microscopy and fluorescence microscopy from SK-MEL-28 culture (A) and after incubation with BM-vesicles labelled with Nile Red (NR) (B), with free NR (positive control) (C), and with BM-vesicles without NR (D). Scale bar = 100 mm.

BM-vesicles labelled with Nile Red (NR), a hydrophobic dye that stains cell membranes and lipids, were applied to SK-MEL-28 cells, and after 30 min, the fluorescence in the cells was checked with fluorescence microscopy (Figure 10). As it can be observed, fluorescence appeared in cells when the fluorescent dye was applied to the cell culture as a positive control (Figure 10(C)), and when it was applied in stained BM-vesicles. Negative fluorescence appeared in control cells (Figure 10(A)) and BM vesicles were applied to the cells without the fluorescent dye (Figure 10(D)).

## Discussion

Nanocarriers obtained from natural sources, for use in therapy and cosmetics, are the focus of many research studies due to their great potential provided by their compatibility with human cells (Singh et al. 2020). Within this group, membrane nanovesicles extracted from plant material have been previously studied, showing their ability to stabilize bioactive compounds, such as glucosinolates (glucoraphanin) through interactions with aquaporins (Martínez Ballesta et al. 2016), with the aquaporins providing stabilization (Martínez Ballesta et al. 2018). Then, the application of this type of vesicles was tested in keratinocytes, which revealed the fusion between human and plant membranes (Yepes-Molina et al. 2020), or the potential to improve the effect of bioactive compounds encapsulated in these vesicles (Yepes-Molina et al. 2021). In addition to applications in the cosmetics industry, research has been conducted on the use of these vesicles in other areas, such as foliar fertilization in agriculture (Rios et al. 2019, 2020). Therefore, in this work, we studied sulforaphane (SFN) encapsulated in broccoli membrane vesicles (BM-vesicles) for melanocyte proliferation inhibition and related genes. The encapsulation of compounds, such as SFN is crucial for their use in pharmaceutical and nutraceutical industries, as it is very unstable in most environments and needs the protection of nanocarriers. SFN has previously been encapsulated with good results in different types of delivery systems [i.e., albumin microspheres (Do et al. 2010), gelatin/gum Arabic, and gelatin/pectin complexes (García-Saldaña et al. 2016), liposomes (Mohanty et al. 2020)].

Physicochemical characterization of a drug delivery system is the first requirement in its design and development. Parameters, such as the mean size, polydispersity index, and zeta potential are crucial for the possible development of encapsulated drugs and delivery systems. In particular, a small vesicle size is required for topical drug delivery systems for skin disorders treatments. Specifically, the existing literature establishes that between 100 and 600 nm nanocarriers would be optimal for a transdermal application (Danaei et al. 2018). Our data from the DLS analyses showed BM-vesicles with a mean size of around 400 nm, with a narrow size distribution ranging from 0.2 to 0.3, which no change when SFN was encapsulated, and therefore our system is suitable for use in these types of applications. Regarding zeta potential, this parameter provides information on physical properties, such as the stability of the formulation. Values of −27 mV, which could be considered stable (Vallar et al. 1999), were measured in BM-vesicles with and without SFN. Also, a negative surface charge is needed for topical drug delivery systems, as negatively charged vesicles can effectively interact with the skin (Ogiso et al. 2001; Gillet et al. 2011). Furthermore, BM-vesicles were spherical in shape, as shown by the transmission electron microscopy analysis, which is key for protecting the integrity of the encapsulated compounds (Do et al. 2010). The osmotic water permeability coefficient (*Pf*) measurement is another way to evaluate the stability and viability or functionality of these types of vesicles when a drug, such as SFN is encapsulated. In accordance with Martínez Ballesta et al. (2018), high *Pf* values were associated with the functionality of vesicles and aquaporins, indicating vesicle integrity. In this sense, our results show no significant changes in the *Pf* values of BM-vesicles when SFN was encapsulated, resulting in the non-significant effect of SNF on physical membrane parameters. Another important parameter in encapsulation studies is entrapment efficiency (EE). The EE of SFN in BM-vesicles was 40%. This percentage is similar to that obtained previously in a plant membrane system with other compounds (Yepes-Molina et al. 2020), or the EE obtained when SFN was encapsulated in a system based on artificial liposomes (Mohanty et al. 2020).

Characterizations to determine the content of isothiocyanates that may remain in the BM-vesicles after the isolation process was carried out. BM-vesicles were isolated from broccoli leaves, which also contain a considerable amount of GLSs (precursors of ITCs) (Liu et al. 2018). GLSs were not detected when BM-vesicles were analyzed by UHPLC-QqQ-MS/MS, although different ITCs were detected (SFN, erucin, iberine, and indole-3-carbinol). The transformation of GLSs to ITCs may be due, on the one hand, to the isolation process of the vesicles, which involves aggressive procedures, such as trituration or ultracentrifugation involving the myrosinase enzyme, responsible for the conversion (Martinez-Ballesta and Carvajal 2015). Myrosinase is usually found as myrosinase grains inside of the vacuole, but there are also indications that it may appear associated with the tonoplast (Lüthy and Matile 1984; Chhajed et al. 2019) and therefore it is possible that BM-vesicles contained some associated myrosinase. In fact, myrosinase was detected in the BM-vesicles proteomic analysis, and its activity in BM-vesicles was measured using sinigrin as the substrate (data not shown). Thus, BM-vesicles act as a nanocarrier, but may also contain some ITCs compounds that could affect the treatment of melanocytes in addition to SFN. In fact, all of the ITCs identified in the BM-vesicles have been previously studied due to their health benefits to human health, as they show antimicrobial, antiviral, antioxidant, and anticancer activities (Mitsiogianni et al. 2019; Singh et al. 2021).

As BM-vesicles are proteoliposomes, the membrane proteins differentiate them from other conventional delivery systems, such as liposome-type nanocarriers. Proteins have been associated with a higher stability of proteoliposomes as compared to other nanocarriers (Martínez Ballesta et al. 2016; Seneviratne et al. 2018), and therefore a BM-vesicles proteomic analysis was carried out. Some of the highlighted molecular functions of BM-vesicles proteins were antioxidant activity or transmembrane transporter activity, which could be interesting in the application of BM-vesicles as nanocarriers. Proteins with antioxidant activity found in BM-vesicles, such as catalase, superoxide dismutase (SOD), peroxidase, or peroxiredoxin, may provide health benefits in addition to those provided by the compounds encapsulated in the vesicles. The antioxidant capacity is altered in different types of cancer cells. For example, a decreased catalase activity (Picardo et al. 1996), some peroxidase enzymes (Chen et al. 2016), and SOD have been found in highly metastatic melanoma cells (Kwee et al. 1991). On the other hand, proteins with transmembrane transporter activity could improve the delivery of encapsulated compounds.

Once the BM-vesicles were characterized, and once it was proven that the encapsulation of SFN did not alter their physicochemical properties, the anticancer activity of SFN-loaded BM-vesicles was investigated in SK-MEL-28 cells, a malignant melanoma cell line. First of all, the cytotoxicity caused in cells by the BM-vesicles was measured. BM-vesicles without any encapsulated drug caused a decrease in cell viability of about 50%. This could be due, on the one hand, to bioactive compounds associated with membrane vesicles, such as some of the identified isothiocyanates, or others that were unidentified, and on the other hand, by the proteins contained in the membranes. As with the ITCs, it is possible that some compounds were retained during the membrane isolation process, and these could have had an effect on the cell viability of SK-MEL-28, which could be an advantage in certain applications of these vesicles. Thus, more research is needed to corroborate this hypothesis. Regarding cytotoxicity associated with SFN, there an increase in dose-dependent cytotoxicity was found, in agreement with other published studies. A low dose (5–10 µM) increased the cell viability (Cristiano et al. 2019), an intermediate dose (20–30 µM) caused a decrease in cell viability of 30–40% (Do et al. 2010; Rudolf et al. 2014; Soni et al. 2018), and high doses entailed cytotoxicity of about 70–80% (Rudolf et al. 2014; Soni et al. 2018). SFN has been shown to have anticancer activity, as SFN induces apoptosis in pre-cancerous and tumour cells of melanoma and other types of cancers. However, in addition to being therapeutic, SFN has also been shown to act as a preventive agent (Cornblatt et al. 2007). As for the effect of BM-vesicles with SFN, differences with respect to the cytotoxicity caused by BM-vesicles were shown when an intermediate concentration of SFN (25 µM) was encapsulated. A decrease in cell viability in comparison with free SFN and BM-vesicles revealed a synergistic effect as revealed by Cristiano et al. (2019), when SFN loaded in ethosomes were applied to SK-MEL-28 cells. The ability of BM-vesicles to increase the anticancer activity of the SFN is probably due to their fusion with the cell membranes, resulting in the direct drug release into the cytoplasm (Godin and Touitou 2004). In this sense, the fusion capacity of our vesicles was already verified in a previous work carried out with keratinocytes (Yepes-Molina et al. 2020).

In general, the anticancer properties of SFN have been described in several *in vitro* and *in vivo* studies and appeared to involve multiple intracellular mechanisms of action (Su et al. 2018). Nonetheless, there are molecular details about the action of SFN that have yet to be revealed. In this work, it was investigated whether encapsulated SFN acted in a similar way to free SFN, basing the analyses on genes previously described as key genes in the mechanisms of action of SFN. TNFα is a pro-inflammatory cytokine that is important in apoptosis, and altered levels of expression of this cytokine have been observed in different tumours, including melanoma (Lázár-Molnár et al. 2000). Previous studies indicated that the use of SFN was adequate treatment for decreasing the altered levels of SFN (Hamsa et al. 2011), but our results did not show significant differences in TNFα gene expression as compared to untreated cells. This may be due to our cell line (SK-MEL-28) being a highly metastatic melanoma cell line (Kim et al. 2017), and data showed by Arcidiacono et al. (2018) suggested that metastatic lines are more resistant to SFN treatment. Regarding p53, a pro-apoptotic gene, SFN up-regulated its expression, displaying an antitumor effect by induction of either cell-growth arrest or apoptosis (Rudolf et al. 2014). Our results, according to previous studies (Hamsa et al. 2011; Rudolf et al. 2014), showed p53 gene up-regulation after free SFN treatment at two tested concentrations (5 and 25 µM). In the case of SFN encapsulated in BM-vesicles, no significant differences were observed with respect to untreated cells. This could be related to slow and controlled delivery of SFN from BM-vesicles. On the other hand, p53 activates other pro-apoptotic proteins, such as BAX. BAX was up-regulated with the BM-vesicles treatment, which could have been triggered by plant compounds associated with the membranes, as well as lipids and proteins. Some compounds present in plant cell membranes, such as carotenoids have exhibited pro-oxidant actions that trigger apoptosis of cancer cells through enhanced reactive oxygen species (ROS) generation, which entails the regulation of key pro and anti-apoptotic genes, for example, with the up-regulation of BAX gene (Shin et al. 2020).

A clearly different expression pattern of AQP3 was found when SFN was applied either free or encapsulated. Treatments with BM-vesicles up-regulated AQP3 expression, which also occurred with the SFN 25 µM treatment. Helwa et al. (2017), in a recent study, showed that SFN, an Nrf2 activator, increased AQP3 levels and suggested that AQP3 expression may be regulated by Nrf2 and that up-regulated AQP3 expression inhibits proliferation. Also, the activation of Nrf2 up-regulated antioxidant systems (Bai et al. 2015), which could help with a favourable prognosis of cancer, due to melanoma cells exhibiting increased oxidative stress, which damage surrounding tissue and allow the progression of metastasis (Sander et al. 2003). Thus, the treatment applied, including BM-vesicles with SFN, up-regulates AQP3 expression, which correlated with the decrease in SK-MEL-28 cell viability with these treatments. Nevertheless, AQP3 has been shown to play a role in the migration of some cancer cells (Marlar et al. 2017), which could be contradictory, as SFN (an Nrf2-Stimulator) is a potential anti-carcinogenic compound. No previous studies were found where aquaporins, such as AQP3 were analyzed after an SFN treatment of a cancer cell culture. This should be considered when finding adequate doses for an SFN treatment, as this compound could be profitable in cancer treatment if certain molecular markers are measured, although it could have an effect on other genes or pathways that could negatively influence the progression of cancer. AQP3 is crucial in many biological processes and is essential in skin homeostasis (Bollag et al. 2020). Thus, it must be under the spotlight in these types of studies, especially when it comes to skin cancer.

On the other hand, the levels of SFN and its derivative (SFN-CYS) were measured in SK-MEL-28 after treating the cells with two concentrations of free SFN and SFN-loaded in BM-vesicles for 24 h. The concentration of SFN was measured both in cell lysates and in the culture medium. When the amounts of SFN applied were low, a clear action of the BM-vesicles was observed, improving the entry of SFN into cells. This, as already mentioned before, maybe due to the fusion of BM-vesicles with the outer cell membranes (Yepes-Molina et al. 2020). Indeed, these results were in accordance with data obtained in the analysis of SFN in the cell culture medium, as there was less SFN in the medium when it was applied in its encapsulated form. In contrast, the application of a high concentration of SFN did not reveal differences in the amount of SFN inside of the cells when SFN was applied free or encapsulated, although a lower amount of SFN remained in the culture when SFN was applied encapsulated in BM-vesicles. Cells in culture are highly accessible and can easily absorb compounds added to the medium, and perhaps 25 µM SFN is a high enough quantity so as not to present problems entering the cells. Even so, indirectly, we can determine that with BM-vesicles, a greater quantity entered the cells, as a smaller amount remained in the medium, and perhaps it was also metabolized faster by these cells. SFN-CYS is an intermediate compound of SFN metabolism via the mercapturic acid pathway and had been detected inside of melanoma cells, which reveal the ability of the cancerous cell to metabolize SFN. Indeed, recently SFN-CYS was shown as a potential drug against different types of cancers (Lin et al. 2017; Zhou et al. 2020).

## Conclusions

We have shown that BM-vesicles could serve as nanocarriers for drugs, such as SFN, by demonstrating the suitability of the system with deep physicochemical characterization and the measurement of anticancer activity in melanoma cells. The results of this deep characterization of BM-vesicles revealed that these vesicles contained ITCs and proteins associated with an antioxidant activity, which could be beneficial in biotechnological applications. Also, the results of the cell culture assays showed, on the one hand, better absorption of SFN into the cells when it was encapsulated in BM-vesicles and the metabolism of SFN by melanoma cells as we were able to detect SFN-CYS inside of cells, which provided further evidence of the anticarcinogenic effect of the applied treatment. And on the other hand, the analysis of some common cancer molecular markers revealed a reduction of cancer cell markers after the treatment with these vesicles. Furthermore, the fact that AQP3 increased in expression could be important in melanocytes studies. In this way, further and more in-depth research is necessary to advance in this matter.
